# Protective Properties of FOXO1 Inhibition in a Murine Model of Non-alcoholic Fatty Liver Disease Are Associated With Attenuation of ER Stress and Necroptosis

**DOI:** 10.3389/fphys.2020.00177

**Published:** 2020-03-11

**Authors:** Hao-ran Ding, Zhen-ting Tang, Ning Tang, Zheng-yi Zhu, Han-yi Liu, Chen-yan Pan, An-yin Hu, Yun-zhen Lin, Peng Gou, Xian-wen Yuan, Jia-hui Cai, Chun-long Dong, Jing-lin Wang, Hao-zhen Ren

**Affiliations:** ^1^Department of Hepatobiliary Surgery, Affiliated Drum Tower Hospital of Nanjing University Medical School, Nanjing, China; ^2^Department of Hepatobiliary Surgery, Nanjing Drum Tower Hospital Clinical College of Nanjing Medical University, Nanjing, China; ^3^Department of Hepatobiliary Surgery, Nanjing University of Chinese Medicine, Nanjing, China; ^4^Department of Pediatrics, Wuxi People’s Hospital Affiliated to Nanjing Medical University, Wuxi, China

**Keywords:** non-alcoholic steatohepatitis, FOXO1, endoplasmic reticulum stress, necroptosis, NAFLD (non alcoholic fatty liver disease)

## Abstract

**Aim:**

The pathogenesis of non-alcoholic fatty liver disease is currently unclear, however, lipid accumulation leading to endoplasmic reticulum stress appears to be pivotal in the process. At present, FOXO1 is known to be involved in NAFLD progression. The relationship between necroptosis and non-alcoholic steatohepatitis has been of great research interest more recently. However, whether FOXO1 regulates ER stress and necroptosis in mice fed with a high fat diet is not clear. Therefore, in this study we analyzed the relationship between non-alcoholic steatohepatitis, ER stress, and necroptosis.

**Main Methods:**

Male C57BL/6J mice were fed with an HFD for 14 weeks to induce non-alcoholic steatohepatitis. ER stress and activation of necroptosis in AML12 cells were evaluated after inhibition of FOXO1 in AML12 cells. In addition, mice were fed with AS1842856 for 14 weeks. Liver function and lipid accumulation were measured, and further, ER stress and necroptosis were evaluated by Western Blot and Transmission Electron Microscopy.

**Key Findings:**

Mice fed with a high fat diet showed high levels of FOXO1, accompanying activation of endoplasmic reticulum stress and necroptosis. Further, sustained PA stimulation caused ER stress and necroptosis in AML12 cells. At the same time, protein levels of FOXO1 increased significantly. Inhibition of FOXO1 with AS1842856 alleviated ER stress and necroptosis. Additionally, treatment of mice with a FOXO1 inhibitor ameliorated liver function after they were fed with a high fat diet, displaying better liver condition and lighter necroptosis.

**Significance:**

Inhibition of FOXO1 attenuates ER stress and necroptosis in a mouse model of non-alcoholic steatohepatitis.

## Introduction

Obesity and its related metabolic syndrome have gradually become an important factor affecting human health all over the world. The incidence of NAFLD in western countries has reached 30%, which has seriously affected worldwide healthcare (Abdelmalek, 2016; Younossi et al., 2016; Fiorucci et al., 2018). NAFLD is characterized by excessive fat deposition in hepatocytes caused by other definite liver-damaging factors besides alcohol and can progress to non-alcoholic steatohepatitis, liver cirrhosis and hepatocellular carcinoma (Qian et al., 2019). Thus, the treatment of NAFLD is a focus of world health concerns. The treatment of NAFLD is mainly based on diet control and exercise. Currently, medical treatment of non-alcoholic steatohepatitis lacks the effects of properly designed drugs and equipment (Brunt et al., 2015), in part because, at present, the mechanism of non-alcoholic fatty liver disease is not completely clear. A widely accepted theory regarding the pathogenesis of NAFLD is the “Two Hits” theory (Cheung et al., 2019). The first hit is insulin resistance caused by fat accumulation, and the second hit is lipid peroxidation, oxidative stress, and secondary inflammation. Oxidative stress, including mitochondrial dysfunction and ER stress, is the main cause of cell injury and death in the disease.

The endoplasmic reticulum is an important organelle in eukaryotic cells, a cavity surrounded by a closed membrane system and another membrane, forming a three-dimensional network structure that can communicate within (Fernandez et al., 2015; Oakes and Papa, 2015). Changes in calcium homeostasis and accumulation of unfolded or misfolded proteins in the endoplasmic reticulum can trigger ER stress (Lebeaupin et al., 2018). Proper folding of proteins after translation requires the involvement of other proteins in the endoplasmic reticulum. Among them, the glucose regulatory protein GRP78 plays a key role in the UPR process (Ogawa et al., 2017; Gifford and Hill, 2018). GRP78 is also known as BIP. Up-regulation of GRP78 expression is a hallmark of UPR and ER stress. Prolonged or chronic ER stress without adaptive response will eventually lead to apoptosis, where CHOP is an important pro-apoptotic protein in UPR (Wang and Kaufman, 2016; Wu et al., 2016). NAFLD lipid sources include residues of chylomicrons in the diet, release of free fatty acids of triacylglycerols in adipose tissue, and newly synthesized adipose tissue. Excessive lipid accumulation and insulin resistance are the main causes ER stress, therefore, regulating ER stress is important in alleviating the progression of NAFLD. Because ER stress plays an important role in lipid metabolism, it has become a focal point in the research of non-alcoholic steatohepatitis (Kanda et al., 2018; Wang et al., 2018).

Necroptosis is a recently discovered cell death pathway and is thought to be involved in the development of many diseases (Gunther et al., 2013; Karunakaran et al., 2016; Mizumura et al., 2016; Dhuriya and Sharma, 2018). Necroptosis is a form of cell death that lies between necrosis and apoptosis. Receptor-interacting protein3 (RIP3) is a switch molecule for cell necroptosis, whereby the recruitment of RIP3 leads to recruitment and phosphorylation of MLKL, eventually leading to changes in cell membrane permeability, which can eventually result in cell membrane rupture (Christofferson and Yuan, 2010). Necroptosis can be regulated by TNFα and toll-like receptors 3 and 4 (TLR3/TLR4). Hepatocyte death due to hepatic steatosis can cause inflammation, fibrosis and other liver problems, and it is closely related to the aggravation of NAFLD. Reducing hepatocyte death is critical to reversing the progression of NAFLD. Roychowdhury found that a high-fat diet increased the level of necroptosis in liver. Necroptosis is closely related to inflammation and the accumulation of fat in the liver, but the specific mechanisms are not clear (Roychowdhury et al., 2016). At present, there are few studies on necroptosis in the field of NAFLD. So, we carried out research with the aim of gaining insight into the phenomenon and mechanism of necroptosis in NAFLD.

The forkhead family is a family of highly functional transcription factors with highly conserved DNA binding domains (Wang et al., 2016), they are key downstream factors in the insulin/IGF-1 signaling pathway and play an important role in regulating cell growth, differentiation, and metabolic regulation. FOXO1 has been shown to be involved in the regulation of glucose metabolism. Phosphorylated AKT phosphorylates FOXO1 and moves from the nucleus to the cytoplasm, restricting FOXO1 transfer into the nucleus, and subsequently reducing its transcriptional activity (Kim et al., 2011). In recent years, there has been more research into the regulation of hepatic lipid metabolism by FOXO1 (Matsumoto et al., 2006; Liu et al., 2016; Li et al., 2017). Decreased FOXO1 expression in insulin receptor knockout mice has been shown to lead to lower lipid deposition in the liver (Matsumoto et al., 2007), which suggests that FOXO1 may be regulate NAFLD progression. As mentioned above, necroptosis and ER stress are involved in the progression of NAFLD. As an important regulatory protein for cell survival and metabolism, FOXO1 may be an important regulatory factor in necroptosis and ER stress in NAFLD. In this study we aimed to investigate whether FOXO1 participates in ER stress and necroptosis during the progression of NAFLD. We explored the relationship between FOXO1 and ER stress and necroptosis in NAFLD, and we investigated the effect of inhibition of FOXO1 on these processes, thus hoping finding a therapeutic target for NAFLD. Our hypothesis is that there is a link between necroptosis and ER stress in NAFLD, and this is our focus.

## Materials and Methods

### Western Blotting Analysis

Western blotting analysis was performed according to the standard protocols (Machado et al., 2015). Protein was extracted from AML12 cells or liver with RIPA lysis buffer and quantified using a BCA protein quantitative kit. Equal amounts of protein were separated on 12% glycine SDS-PAGE gels and transferred to a PVDF membrane. Membranes were blocked in 7% dry milk in TBST for 1 h before being incubated in the indicated primary antibodies. Primary antibodies were rabbit FOXO1 (Abcam, ab39670), p-FOXO1 (Abcam, ab131339), GRP78 (Abcam, ab21685), PERK (Cell signal Technology, #3192), CHOP (Cell signal Technology, #2895), RIP1 (Cell signal Technology, #3493), RIP3 (Santa-Cruz, sc-374639), and p-MLKL (Abcam, ab196436). GAPDH (Abcam, ab181603) expression was used as a loading control. The immunoreative bands were visualized with enhanced chemiluminescence (ECL) reagent (Thermo Fisher Scientific, Waltham, MA, United States). Densitometric analysis of signal intensity was performed using Image J software (NIH, Bethesda, MD, United States).

### Animals

Male C57BL/6J mice aged 4 weeks were obtained from animal experimental base of Nanjing Drum Tower Hospital Affiliated to Nanjing University. Mice were allowed free access to sterile water and Research Diet 12492 (60 kcal%). This experiment passed the approval of the institutional animal care and use Committee of Nanjing University.

### Experimental Groups

A total of 36 mice were randomly divided into three groups: a standard chow diet (CFD) group; a 60 kcal% high fat diet group (HFD) group; and a high fat diet group treated with the FOXO1 inhibitor AS1842856 by gavage (20 mg/kg) (HFD + AS). Male C57BL/6J mice were fed with high fat diet (HFD: 60% fat, 20% protein, and 20% carbohydrates; 520 kcal/100 g; D12492; Research Diets, New Brunswick, NJ, United States) for 14 weeks to induce steatosis.

### Liver Function Examination

Levels of ALT, AST, and triglycerides and cholesterol were determined with an automated biochemical analyzer (iMagic-M7; Mindray, Shenzhen, China).

### Histology Analysis

Liver samples were immobilized with 4% paraformaldehyde and then dehydrated. They were embedded in paraffin and then made into slices (<3 μm thick, three from each liver). The pathological examination was performed using hematoxylin and eosin staining.

### Transmission Electron Microscopy

Liver specimens were collected, fixed and solidified according to the routine method. Specimens were adhered to the slices after drying naturally. Samples were then observed using TEM. Thickness of the sections applied for TEM analysis is 40 nm. We chose 400 mesh copper as the grids for TEM (Transmission Electron Microscope, JEM-1011, Japan).

### PI Staining

AML12 cells were cultured in growth medium containing high-glucose Dulbecco’s modified Eagle’s medium (DMEM, Gibco, Grand Island, NY, United States) supplemented with 10% fetal bovine serum (Sciencell, San Diego, CA, United States), 100 mg/mL streptomycin and 100 U/mL penicillin. The cells were cultured in a humidified atmosphere containing 5% CO_2_ at 37°C. AML12 cells were treated with PA (0.5 mM) for 24 h. AML12 cells were immobilized in 4% paraformaldehyde for 30 min and washed twice with PBS. After treatment with RNase A for 30 min at 37°C. 10 mg/mL propidium iodide (PI, KeyGEN BioTECH, China) was used to stain cell nuclei. After counterstaining with DAPI, we observed the cells using fluorescence microscopy.

### Immunofluorescence

AML12 cells were treated with 0.3% Triton (Triton X-100, Dilution with PBS) for 15 min. 10% Normal goat serum was dripped on to the slide, which was then sealed at room temperature for 30 min. The slides were then incubated overnight in the primary antibodies at 4°C. PBST was used to wash three times before incubation with second antibody for 30 min at room temperature. The nuclei were stained with DAPI and the following antibodies were used to highlight other cellular components: anti-RIP3 (Santa-Cruz, sc-374639), goat antimouse IgG H&L (Alexa Fluor^®^ 488) (Abcam, ab150117). 12 mice were examined in each group, and 3 independent view fields per section were quantified.

### Statistical Analysis

All data were represented by the mean ± standard deviation. The comparison of two independent samples performed using a Student’s *t*-test. In statistical analysis we used “^∗^” to represent *P* < 0.05. Prism software was used for all statistical analysis.

## Results

### Levels of FOXO1, ER Stress and Necroptosis Related Proteins Increase in HFD Fed Mice

**FIGURE 1:**
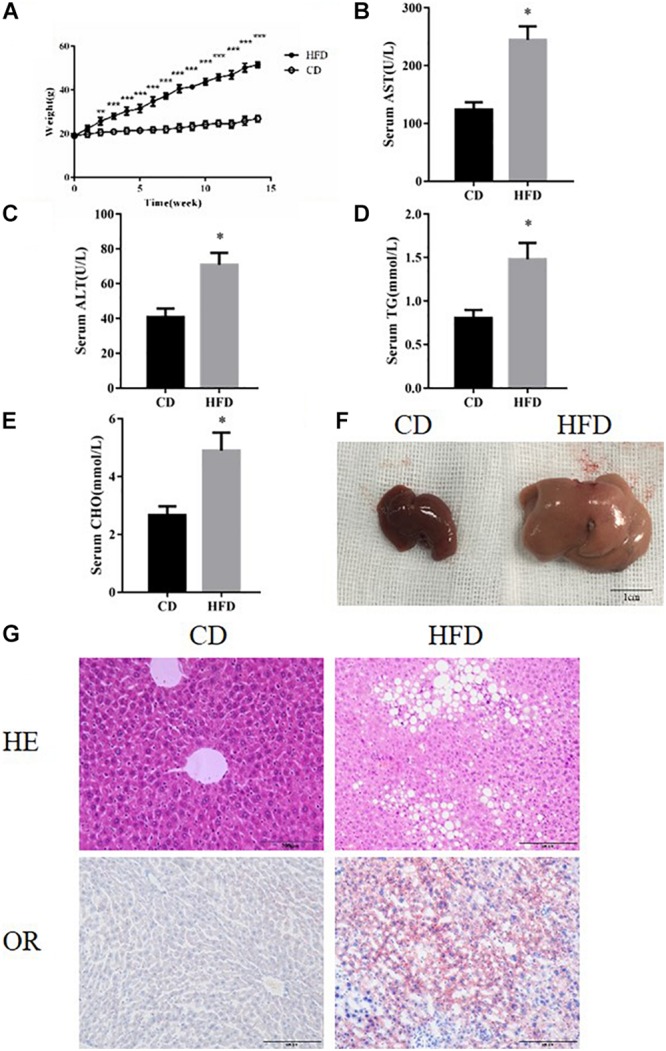
HFD feeding established NAFLD models. **(A)** Body weight of HFD and CD feeding mice. **(B–E)** The levels of serum TG, AST, ALT, and CHO in CD and HFD feeding mice. **(F)** Representative images of liver after CD or HFD feeding. Scale bars: 1 cm. **(G)** Representative H&E staining and Oil red staining of liver sections after CD or HFD feeding for 14 week. ^∗^*P* < 0.05 vs. CD controls. *t*-Test, data are shown as mean ± standard deviation.

**FIGURE 2:**
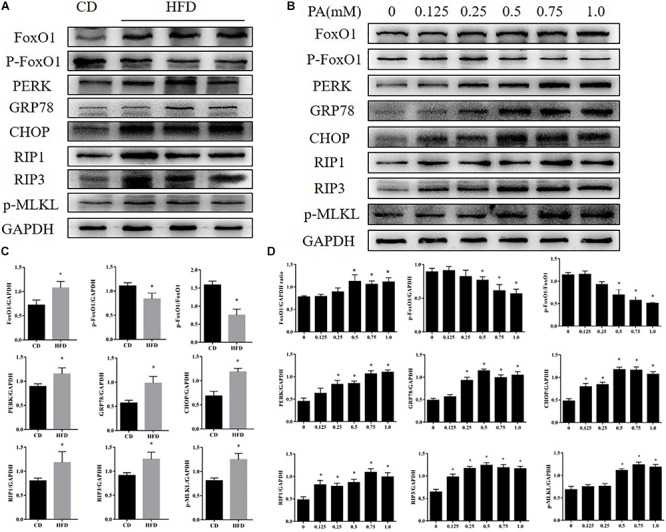
Effects of PA on FOXO1, ER stress, and necroptosis in AML12 cells. **(A)** Immunoblot analysis of FoxO1, p-FoxO1, PERK, GRP78, CHOP, RIP1, RIP3, p-MLKL in CD and HFD feeding mice. **(B)** Immunoblot analysis of FoxO1, p-FoxO1, PERK, GRP78, CHOP, RIP1, RIP3, p-MLKL in cells treated with PA. **(C)** Quantitative analysis of **(A)**. **(D)** Quantitative analysis of **(B)** ^∗^*P* < 0.05 vs. CD controls. *t*-Test, data are shown as mean ± standard deviation.

Compared with the control group, mice fed with the HFD developed typical NAFLD characteristics and displayed worse liver function, observed using the following measurements; body weight, serum triglycerides, cholesterol and serum ALT and AST (Figures 1A–E). By comparing liver specimens from the mice, we found that the liver of mice fed with the HFD was larger and more yellow then the control group (Figure 1F). HE and Oil Red staining of liver sections from HFD fed mice exhibited lipid accumulation and cell vacuolization (Figure 1G). Previous studies have shown that the expression of FOXO1 is linked with dysfunction in glucose and lipid metabolism (Al-Massadi et al., 2019). To investigate the effect of FOXO1 on NAFLD mice, the expression of FOXO1 was analyzed in HFD fed mice by western blot. We found that the expression of FOXO1 was significantly increased in the HFD fed mice compared with the normal mice, while the levels of phosphorylated FOXO1 was significantly decreased in normal mice. Overall, the ratio of p-FOXO1/FOXO1 was significantly increased in HFD fed mice compared with normal mice. In addition, the expression levels of ER stress related proteins (PERK, GRP78, CHOP) were analyzed. The results showed that there was a significant increase in expression of ER stress markers in HFD fed mice. Next, we investigated whether necroptosis occurred in the HFD fed mice. The results showed that expression levels of RIP1, RIP3 and phosphorylated MLKL proteins were all increased in HFD fed mice compared with normal mice (Figures 2A,C).

### Effects of PA on FOXO1, ER Stress and Necroptosis Related Proteins Expression in AML12 Cells

To further explore the changes in FOXO1 levels, ER stress and necroptosis in NAFLD we treated AML12 cells with palmitic acid (PA) to induce an *in vitro* model of NAFLD. AML12 cells were treated with PA (0, 0.125, 0.25, 0.5, 0.75, 1.0 mM) for 24 h. We investigated the impact of PA on ER stress, a cellular response that is closely related to altered metabolism in NAFLD, in AML12 cells. As shown in Figure 2B, GRP78, CHOP and protein kinase R-like ER kinase (PERK), which are major ER stress markers, were significantly up-regulated as the concentration of PA treatment increased. We next assessed whether PA treatment activated necroptosis in AML12 cells, a novel process of caspase-independent cell death (Galluzzi et al., 2017; Shan et al., 2018). We found that protein expression of RIP1, RIP3 and phosphorylated MLKL was strongly increased in the treatment group when compared with the control group. Furthermore, protein levels of FOXO1 were significantly up-regulated in AML12 cells treated with PA. Meanwhile, phosphorylation levels of FOXO1 were significantly down-regulated in AML12 cells treated with PA (Figures 2B,D). We found the same phenomenon in primary hepatocytes (Supplementary Figures S1A,B). Thus, the data indicate that PA treatment activates ER stress, induces necroptosis and increases FOXO1 activity.

### Effects of Inhibition of FOXO1 in AML12 Cells Treated With PA

**FIGURE 3:**
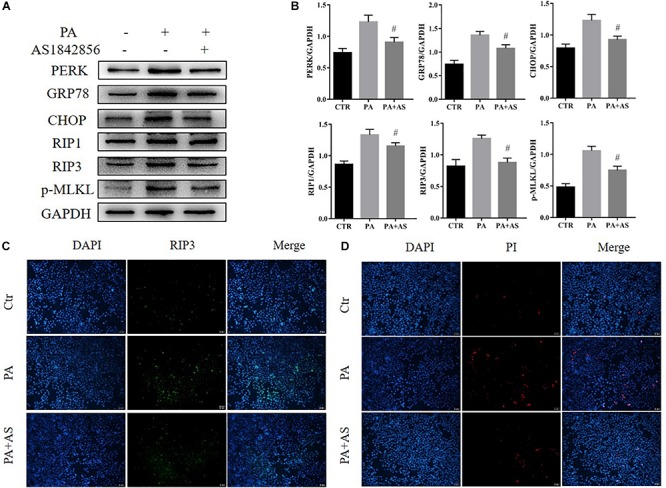
Effects of inhibition of FOXO1 in AML12 cells treated with PA. **(A)** Immunoblot analysis of PERK, GRP78, CHOP, RIP1, RIP3, p-MLKL. **(B)** Quantitative analysis of **(A)**. **(C)** Representative immunofluorescence staining of RIP3 was performed in Ctr, PA, PA + AS. **(D)** Representative immunofluorescence staining of PI. #*P* < 0.05 vs. PA controls. *t*-Test, data are shown as mean ± standard deviation.

To determine whether ER stress and necroptosis could be attenuated via inhibition of FOXO1, we treated AML12 cells with a FOXO1 antagonist (AS1842856, 1 μM, Selleck Chemicals) for 2 h before stimulation with PA (500 μM, Sigma-Aldrich). After 24 h, we detected changes in the activation of ER stress and necroptosis. The results showed that inhibition of FOXO1 suppressed protein expression of GRP78, CHOP, and PERK in AML12 cells (Figures 3A,B). Therefore, we show that inhibiting FOXO1 activity in NAFLD can inhibit ER stress. Next, we wanted to explore the effect of FOXO1 inhibition on necroptosis. Our results showed that RIP1, RIP3 and phosphorylated MLKL were significantly down-regulated in AML12 cells treated with the FOXO1 antagonist in comparison to AML12 cells treated with PA alone (Figures 3A,B). We found that inhibiting the activity of FOXO1 reduces apoptosis levels. Meanwhile, levels of the necroptosis marker protein RIPK3 was significantly lower in the PA + AS group when compared to the PA group (Figures 3C,D and Supplementary Figures S1C,D). Overall, inhibition of FOXO1 can reduce ER stress and necroptosis *in vitro*.

### Effects of Inhibition of FOXO1 in Mice Fed With a High Fat Diet

**FIGURE 4:**
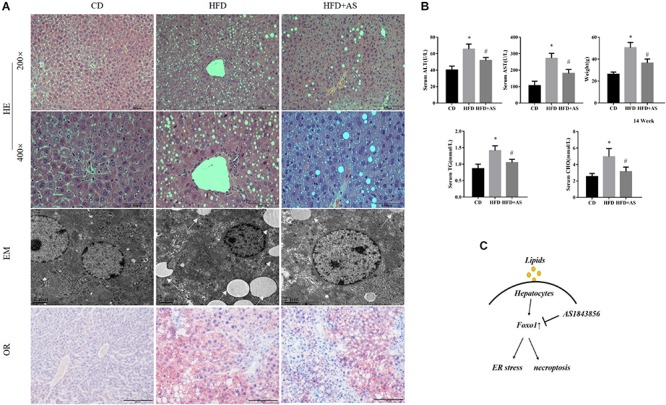
Effects of inhibition of FOXO1 in mice fed with a high fat diet. **(A)** Representative H&E staining, Oil red staining and Transmission electron microscopy of liver sections after CD or HFD or HFD + AS. **(B)** Weight, TG, AST, ALT, and CHO in CD, HFD, HFD + AS. **(C)** Graphical abstract. #*P* < 0.05 vs. HFD controls, ^∗^*P* < 0.05 vs. CD controls. *t*-Test, data are shown as mean ± standard deviation.

To further confirm the role of FOXO1 in NAFLD, C57BL/6J mice were fed with a high fat diet (60 kcal% fat), to induce a model of non-alcoholic steatohepatitis, for 14 weeks. We established fatty liver model by feeding 14 weeks and 20 weeks mice respectively. Both methods can induce fatty liver successfully (Supplementary Figure S3). AS1842856 was used when mice were fed with an HFD (Supplementary Figure S2A). HE staining, oil red staining and TEM showed that HFD caused large-scale macrovesicular steatosis. The group treated with the FOXO1 inhibitor had slightly lower steatosis levels, but still exhibited some signs of macrovesicular steatosis (Figure 4A). We evaluated the effect of FOXO1 on NAFLD by body weight, triglyceride, cholesterol, ALT and AST after 14 weeks of feeding the diet (Figure 4B). Consistent with the results of *in vitro* experiments, inhibition of FoxO1 significantly reduced ER stress and necroptosis (Supplementary Figure S2B). The use of FOXO1 inhibitor increased the phosphorylation of PERK and IRE1-alpha, but had little effect on the MLKL total protein (Supplementary Figures S2C,D). We show unequivocally that inhibition of FOXO1 in mice fed with the HFD improved ER stress and necroptosis (Figure 4C).

## Discussion

It has been shown that unhealthy diet and metabolic dysfunction can lead to NAFLD. Sustained ER stress and necroptosis caused by excessive accumulation of lipids bring about chronic injury of hepatic parenchymal cells, which are the main functional cells in the liver. With this being the case, it is very important to search for the key regulatory factors of ER stress and necroptosis in NAFLD, in order to find a potential treatment for ameliorating NAFLD. We fed an HFD to mice and stimulated AML12 cells with PA to establish an NAFLD model and found that ER stress and necroptosis was enhanced, which was consistent with previous study (Zhang et al., 2017). Further, we found that an important regulatory protein, FOXO1, was associated with ER stress and necroptosis.

Hepatocytes ER stress plays an important bridging role in inflammation, injury, repair, fibrosis and even canceration, and is closely related to the occurrence and progression of NAFLD. ER stress interferes with lipid synthesis and transport while inhibiting lipid secretion from liver cells. The unfolded protein response is insufficient to cope with liver lipotoxicity as lipid accumulation increases and subsequently ER stress is induced. There is increasing evidence that ER stress plays an essential role in promoting the progression of NAFLD (Leamy et al., 2013; Ashraf and Sheikh, 2015; Ao et al., 2016; Xu et al., 2017), and when ER stress initiated by intracellular calcium imbalance and/or accumulation of unfolded protein, GRP78, PERK and CHOP are upregulated, initiating the unfolded protein response. Sustained or excessive ER stress makes the cell’s self-repairing ability insufficient in resisting external stimuli, leading to activation of an ER overload response. Our study found that levels of ER stress-related proteins (GRP78, CHOP, and PERK) were increased with the accumulation of lipid, which was consistent with levels of hepatic steatosis.

Previous studies have suggested that cell necrosis is an irreversible process. The discovery of necroptosis has changed this traditional view and is of great significance to the study of life science and the treatment of disease. Necroptosis is a form of cellular death with necrotic morphological features. Necroptosis is caspase independent, and is a regulated death mode that differs from apoptosis (Radi et al., 2014; Van et al., 2017). Necroptosis is thought to play an important role in liver disease (Afonso et al., 2015) and RIP3 is a key molecule regulating the process. Meanwhile, MLKL is a protein important in stimulating cell membrane rupture (Degterev et al., 2008; Sun et al., 2012). Gautheron et al. (2014) have confirmed that RIP3-dependent necroptosis regulates NAFLD-induced liver fibrosis, and our research shows that increases in the levels of markers of necroptosis (RIP1, RIP3, and p-MLKL) can be induced by the NAFLD mouse model. In future study, we would aim to find targets that can influence the degradation of RIP1 and RIP3 proteins, or the phosphorylation of MLKL, which would thereby interfere with the occurrence of necroptosis.

The forkhead transcription factor (FOXO1) is the first transcription factor found in the FoxO family. Kamo et al. (2013) have previously elucidated that a specific PI3K blockade inhibits Akt/β-catenin signaling and increases FOXO1-mediated TLR4-driven local inflammation. Our data shows that with the accumulation of lipids, protein expression of FOXO1 is significantly increased, while levels of phosphorylated FOXO1 decrease. The transcriptional activation of FOXO1 increased during lipid accumulation in the liver, therefore, we aimed to investigate the role of FOXO1 in regulating NAFLD. Further to this, inhibition of FOXO1 ameliorated lipid-induced ER stress and necroptosis. However, when continuing feeding an HFD and treating with a FOXO1 inhibitor, mice showed lower levels steatosis. Interestingly, Wang and Ren (2016) theorize that the FOXO1 inhibitor AS1842856 acts through phosphorylation. However, Nagashima et al. (2010) found that AS1842856 selectively inhibited FOXO1 without altering the phosphorylation of FOXO1. In this study, we inhibited the function of FoxO1 through inhibitors rather than gene knockout. However in terms of exploring mechanism, it is not as persuasive as gene knockout. In this paper, for HFD induction, other models are not verified. Another recent study conducted in male Swiss mice found no elevation of ER stress after 12 weeks of HFD feeding regimen (de Freitas Carvalho et al., 2019). Future studies using other models of NAFLD (such as CDAA-HFD; Western diet. or other animal species) are needed to confirm our findings (Farrell et al., 2019; Wei et al., 2020).

The inhibition of FoxO1 can block the induction of ER stress and necrosis, and prevent the occurrence of NAFLD. At present, the pathological process of liver fibrosis or NASH induced by high fat diet is still unclear. Some scholars think that hepatic steatosis is an important process in the development of NASH and even hepatic fibrosis. On this basis, our research has certain significance for finding the target of treating fatty liver disease. However, it is still unclear whether inhibition of FoxO1 can reverse the development of NAFLD to NASH or even cirrhosis, which is also the direction of our efforts. We will examine more severe fatty liver disease such as NASH or fibrosis. FOXO family is a transcription regulator. FOXO gene is highly conserved in evolution. There are three highly conserved PKB phosphorylation motifs in its amino acid sequence. Its activity is directly related to phosphorylation state. FOXO can regulate the metabolic balance of cells. It has been previously shown that PERK represents one FOXO regulator (Zhang et al., 2013). But strangely, our study found that FOXO1 can affect the expression of PERK, and PERK can phosphorylate FOXO, which describes an interesting cycle that may be an important regulatory mechanism between FOXO and ER stress.

In future study, more experiments should be conducted to confirm the role of FOXO1 and its relationship with ER stress and necroptosis in NAFLD. Moreover, the specific mechanism between ER stress and necroptosis should be further investigated. In general, we investigated the effects of FOXO1 in regulating steatosis-induced ER stress and necroptosis and our data indicate that FOXO1 does participate in the regulation of NAFLD and that the protein may be a potential therapeutic target in the treatment of NAFLD.

## Data Availability Statement

The datasets generated during the current study are available from the corresponding author on reasonable request.

## Ethics Statement

This study was carried out in strict accordance with the recommendations in the Guide for the Care and Use of Laboratory Animals of the National Institutes of Health. The protocol was approved by the Committee on the Ethics of Animal Experiments of the Nanjing Drum Tower Hospital (Approval No. 20161006). All surgery was performed under isoflurane anesthesia and all efforts were made to minimize animals suffering.

## Author Contributions

HD, ZT, and NT conceived and designed the study, collected and assembled data, performed data analysis and interpretation, and wrote the manuscript. ZZ, HL, CP, AH, YL, PG, XY, JC, and CD conceived and designed the study, collected data, and wrote the manuscript. HR and JW conceived and designed the study, provided financial support and study material, performed data analysis and interpretation, wrote and gave final approval of the manuscript. All authors read and approved the manuscript.

## Conflict of Interest

The authors declare that the research was conducted in the absence of any commercial or financial relationships that could be construed as a potential conflict of interest.

## Supplementary Material

FIGURE S1 Effects of inhibition of FOXO1 in primary hepatocytes treated with PA. **(A)** Effects of PA on FOXO1 and P-FOXO1. **(B)** Immunoblot analysis of PERK, GRP78, CHOP, RIP1, RIP3, p-MLKL. **(C)** Representative immunofluorescence staining of RIP3 was performed in Ctr, PA, PA + AS. **(D)** Representative immunofluorescence staining of PI.

FIGURE S2 Effects of inhibition of FOXO1 on ER stress and necroptosis in mice fed with a high fat diet. **(A)** Body weight of HFD + AS feeding mice. **(B)** Immunoblot analysis of PERK, GRP78, CHOP, RIP1, RIP3, p-MLKL. **(C)** Immunoblot analysis of IRE-1α, P-IRE-1α, PERK, P-PERK, MLKL total protein in *in vitro* experiment. **(D)** Immunoblot analysis of IRE-1α, P-IRE-1α, PERK, P-PERK, MLKL total protein in *in vivo* experiment.

FIGURE S3 Fatty liver model by feeding 14 weeks and 20 weeks. **(A)** Body weight of HFD (14 w) and HFD (20 w) feeding mice. **(B)** Oil red staining of liver sections after HFD feeding for 14w or 20 w.

## Abbreviations

ALT: alanine transaminase
AST: aspartate aminotransferase
BIP: binding immunoglobulin heavy chain protein
CHOP: CCAAT-enhancer-binding protein homology protein
ER stress: endoplasmic reticulum stress
FOXO1: Forkhead box protein O1
GRP78: glucose-regulated protein 78
HFD: high-fat diet
MLKL: mixed lineage kinase domain-like protein
NAFLD: non-alcoholic fatty liver disease
PBS: phosphate buffered saline
RIP1: receptor-interacting protein1
RIP3: receptor-interacting protein3
TC: total cholesterol
TEM: transmission electron microscopy
TGs: triglycerides
TLR3/4: toll-like receptors 3/4
UPR: the unfolded protein response.
