# Supplementary figures and images for: Protective Properties of FOXO1 Inhibition in a Murine Model of Non-alcoholic Fatty Liver Disease Are Associated With Attenuation of ER Stress and Necroptosis
Source: Front Physiol. 2020 Mar 11;11:177. doi: 10.3389/fphys.2020.00177 (PMC7078343; doi:10.3389/fphys.2020.00177)

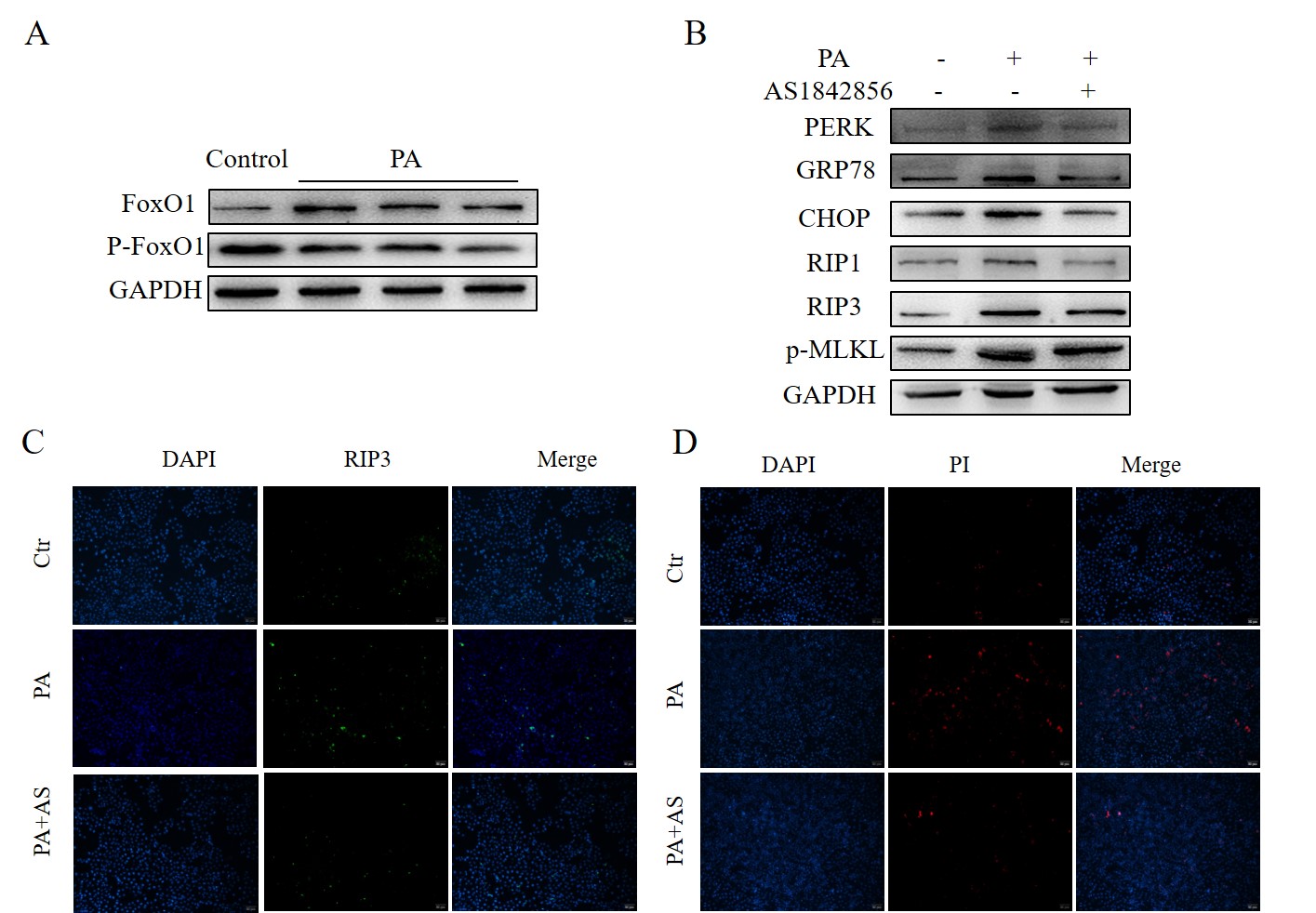

Supplement: FIGURE S1 — Effects of inhibition of FOXO1 in primary hepatocytes treated with PA. (A) Effects of PA on FOXO1 and P-FOXO1. (B) Immunoblot analysis of PERK, GRP78, CHOP, RIP1, RIP3, p-MLKL. (C) Representative immunofluorescence staining of RIP3 was performed in Ctr, PA, PA + AS. (D) Representative immunofluorescence staining of PI. [file Image_1.JPEG]

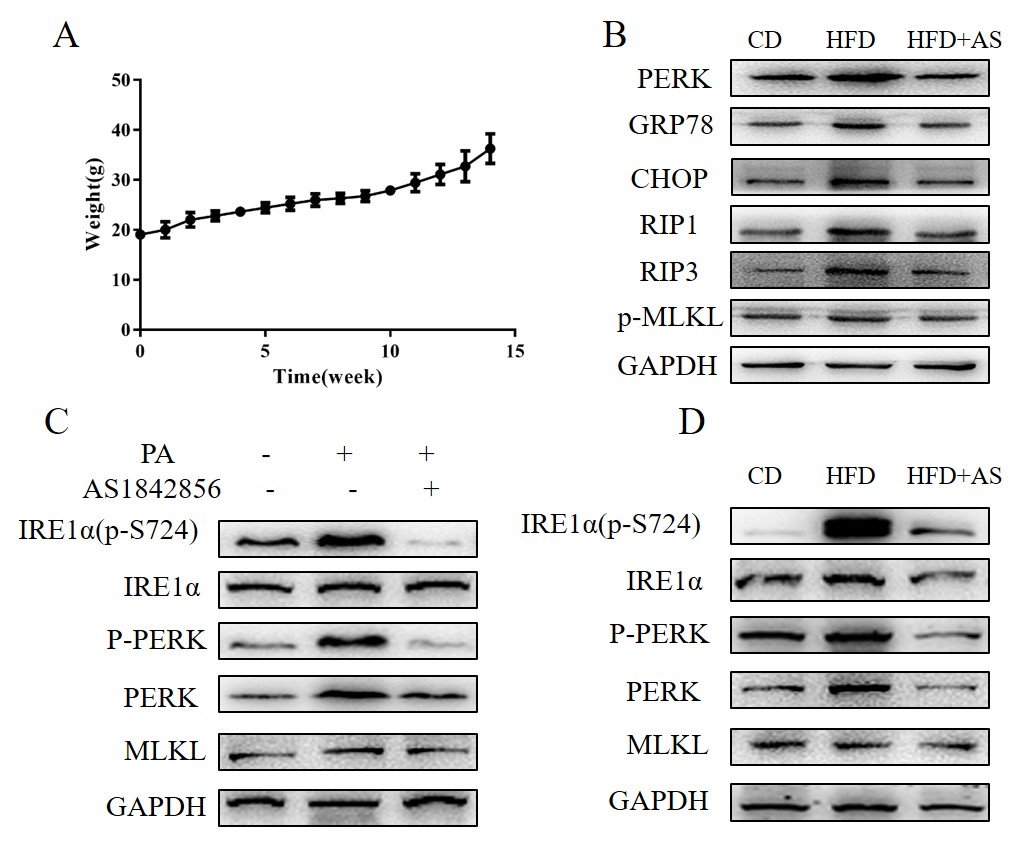

Supplement: FIGURE S2 — Effects of inhibition of FOXO1 on ER stress and necroptosis in mice fed with a high fat diet. (A) Body weight of HFD + AS feeding mice. (B) Immunoblot analysis of PERK, GRP78, CHOP, RIP1, RIP3, p-MLKL. (C) Immunoblot analysis of IRE-1α, P-IRE-1α, PERK, P-PERK, MLKL total protein in in vitro experiment. (D) Immunoblot analysis of IRE-1α, P-IRE-1α, PERK, P-PERK, MLKL total protein in in vivo experiment. [file Image_2.JPEG]

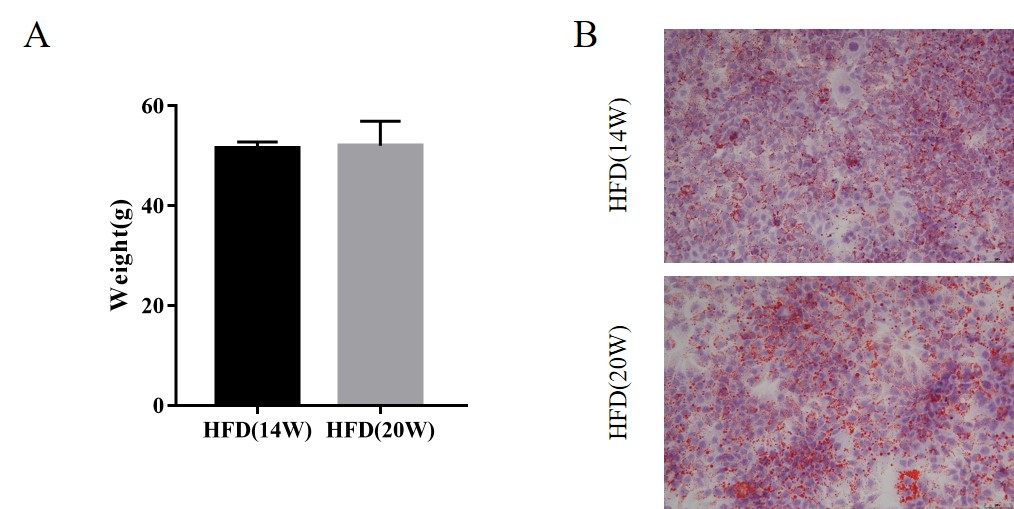

Supplement: FIGURE S3 — Fatty liver model by feeding 14 weeks and 20 weeks. (A) Body weight of HFD (14 w) and HFD (20 w) feeding mice. (B) Oil red staining of liver sections after HFD feeding for 14w or 20 w. [file Image_3.JPEG]
